# On the biophysics of cathodal galvanotaxis in rat prostate cancer cells: Poisson–Nernst–Planck equation approach

**DOI:** 10.1007/s00249-012-0807-7

**Published:** 2012-03-31

**Authors:** Przemysław Borys

**Affiliations:** Department of Physical Chemistry and Technology of Polymers, Section of Physics and Applied Mathematics, Silesian University of Technology, 44-100 Ks. M. Strzody 9, Gliwice, Poland

**Keywords:** Cathodal galvanotaxis, Poisson–Nernst–Planck equation, Mat-Ly-Lu, Motility, Prostate cancer, Metastasis

## Abstract

Rat prostate cancer cells have been previously investigated using two cell lines: a highly metastatic one (Mat-Ly-Lu) and a nonmetastatic one (AT-2). It turns out that the highly metastatic Mat-Ly-Lu cells exhibit a phenomenon of cathodal galvanotaxis in an electric field which can be blocked by interrupting the voltage-gated sodium channel (VGSC) activity. The VGSC activity is postulated to be characteristic for metastatic cells and seems to be a reasonable driving force for motile behavior. However, the classical theory of cellular motion depends on calcium ions rather than sodium ions. The current research provides a theoretical connection between cellular sodium inflow and cathodal galvanotaxis of Mat-Ly-Lu cells. Electrical repulsion of intracellular calcium ions by entering sodium ions is proposed after depolarization starting from the cathodal side. The disturbance in the calcium distribution may then drive actin polymerization and myosin contraction. The presented modeling is done within a continuous one-dimensional Poisson–Nernst–Planck equation framework.

## Introduction

Prostate cancer cells have been investigated for many years by the group of Prof. Djamgoz at Imperial College (Grimes et al. 1995; Siwy et al. 2003; Djamgoz et al. 2001; Mycielska and Djamgoz 2004). One of the most important results concerning these cells is the finding that the voltage-gated sodium channel (VGSC) activity correlates with their metastatic potential. Blocking the VGSCs of the highly metastatic Mat-Ly-Lu cells using tetrodotoxin decreases their metastatic potential, having no effect on the weakly metastatic AT-2 cells (Grimes et al. 1995). Another result, of crucial importance for the presented research, concerns the examination of the motile properties of these cells. Mat-Ly-Lu cells exhibit sensitivity to the external electric field in a phenomenon of cathodal galvanotaxis, which is anodal and much less pronounced in the nonmetastatic AT-2 cells (Djamgoz et al. 2001).

Galvanotaxis was later discovered in further cancer cell lines: the MDA-MB-231 breast cancer line (Fraser et al. 2005; Isbilen et al. 2006) and the A549 lung cancer line (Yan et al. 2009). The lung cancer A549 exhibits cathodal galvanotaxis, as in the case of the Mat-Ly-Lu prostate cancer, while the breast cancer MDA-MB-231 exhibits anodal galvanotaxis, in contrast to Mat-Ly-Lu. This orientation difference will be addressed in the “Results and Discussion” section of the present paper. Apart from cancer cells, galvanotaxis has also been discovered in a number of other cells, as enumerated, for example, by Mycielska and Djamgoz (2004).

**Fig. 1:**
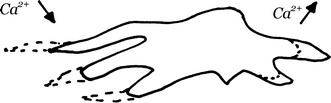
The calcium-mediated crawling process of a cell. A decrease in calcium concentration drives actin polymerization while the increase in calcium reduces polymerization, and causes actin truncation and myosin contraction

The galvanotactic response of a cell makes use of a crawling motion whose molecular mechanism depends on reorganization of the cytoskeleton in response to changes in the internal calcium concentration (Mycielska and Djamgoz 2004; Bray 2001). This consists of four subsequent stages: (1) protrusion growth, (2) protrusion adhesion, (3) cell contraction, and (4) detachment of the rear side of the cell. Protrusion growth occurs due to actin filament polymerization, which is regulated by the protein gelsolin and pH. Gelsolin, in the presence of calcium ions, caps the fast-growing barbed ends of f-actin, inhibiting their polymerization and severing them. Low pH values facilitate actin self-assembly by compensating the anionic character of the monomers (Wang et al. 1989). The contraction of the cell is mediated by myosin, which contracts using adenosine triphosphate (ATP) in the presence of calcium ions (Bray 2001). Within such a framework, directed locomotion may arise after the calcium ion concentration at the rear side of the cell increases, slowing down actin polymerization and causing cell detachment by contraction, while the concentration at the front side decreases, improving actin polymerization (Mycielska and Djamgoz 2004), as illustrated in Fig. 1.

**Fig. 2:**
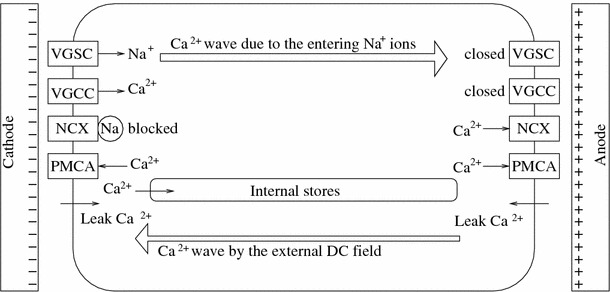
Calcium flow in the cell, relevant for the galvanotaxis of general cells. VGCCs and NCXs do not manifest in Mat-Ly-Lu cells (Djamgoz et al. 2001)

The importance of calcium ions in the motility process leads to consideration of possible calcium pathways into the cytoplasm (Fig. 2): voltage-gated calcium channels (VGCCs), Na^+^/Ca^2+^ exchangers (NCX), plasma membrane Ca^2+^ ATP-ases (PMCAs), leakage channels, internal rearrangements by calcium “waves,” or eventually, calcium release (uptake) from internal stores (Monteith et al. 2007; Carafoli and Brini 2007). It would seem reasonable to expect, for example, that blocking calcium inflow through the VGCCs should have a profound impact on the cellular motion of Mat-Ly-Lu cells. However, this turns out not to be the case, and VGCC activity is not observed in these cells (Djamgoz et al. 2001). Instead, the cellular motion decreases after blockade of VGSCs by tetrodotoxin (TTX) (Djamgoz et al. 2001). A similar effect of VGSC blockade was found in the (anodal) galvanotaxis of MDA-MB-231 breast cancer (Fraser et al. 2005; Isbilen et al. 2006). This finding is consistent with the relation of the VGSCs to the metastatic potential of prostate cancer cells, but the relation between the VGSCs and the cathodal cellular motion remains to be elucidated.

**Fig. 3:**
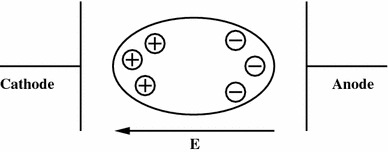
Polarization of ions inside the cell in the presence of an external electric field

One of the classical options for calcium inflow in galvanotaxis (which I critically discuss in the next sections) is through leakage pathways, which are expected to result in a greater contribution to the anodal than to the cathodal part in the presence of a direct-current (DC) field (Cooper and Keller 1984). This would happen because the anodal part of the cell is polarized in the DC field to contain an attractive negative charge while the cathodal part contains a repulsive positive charge (Fig. 3). However, such a mechanism shows no direct relation to the VGSCs, and it remains unclear why it should stop working in Mat-Ly-Lu cells after TTX administration.

This lack of relation to VGSCs could be overcome by considering the NCX exchanger activity (Fig. 2), which stops removing Ca^2+^ from the cell in the presence of Na^+^ (i.e., after VGSC activation) (Hilge et al. 2007). However, NCX does not manifest in Mat-Ly-Lu cells (Djamgoz et al. 2001). Another option could then be pH-driven facilitation of actin polymerization due to pH lowering after the VGSC activity (Carrithers et al. 2007) and reduced calcium release from internal stores due to their sensitivity to pH lowering (Willoughby et al. 2001). However, since the depolarization should eventually spread over the whole cell membrane, this effect could only be transient.

A new effect, supported by informal modeling done on a small amount of discrete ions, was recently proposed by me during the 2008 Gliwice Scientific Meetings (Borys 2008). I suggested that the concentration difference in the Ca^2+^ ions between the cathodal and anodal sides of the cell may result from electrical repulsion of intracellular Ca^2+^ ions by Na^+^ ions entering through the VGSCs on the cathodal side. The VGSCs on the cathodal side should activate more easily compared with the anodal side because of the membrane potential reduction in the presence of a DC field (Fig. 3). Before the depolarization wave can reach the anodal part of the membrane, an electric field gradient builds on the cathodal side, forcing calcium ions to move towards the anode. Then, after the anodal part opens its VGSCs as the membrane depolarization continues, the ions cannot be turned back to the cathode because it already contains a large positive charge (larger than the anodal part). Such an effect also explains the galvanotactic response of the AT-2 cells, which do not upregulate the VGSCs and consequently cannot produce a disturbance in the Ca^2+^ distribution by this mechanism. The importance of timing between the cathode and anode also displays how the NCX activity (in cells other than Mat-Ly-Lu) and VGSC-mediated pH interactions can become directional.

In the present research, the idea of Ca^2+^ repulsion by entering Na^+^ ions is investigated in a formal continuous model using the one-dimensional Poisson–Nernst–Planck (PNP) equation approach. The coefficients for this equation (diffusion coefficients, concentrations) were found through a literature search. The boundary conditions mirror the physical DC field. The model system initially spends some time at rest to obtain ionic equilibrium, then Na^+^ ions are injected to the cathodal compartment, mimicking depolarization. Calcium propagation curves are obtained, revealing an increase in the anodal concentration and a decrease in the cathodal concentration.

## Methods

### The PNP equation

The problem of ionic diffusion is complex because, whenever the ion concentration changes, the electric field distribution changes as well. The electric field in turn influences the forces acting on the ions. Such a process cannot be described by normal diffusion. A drift–diffusion model is required, which implements the forcing of the ions by the electric potential. The mass flux in the drift–diffusion model in its simplest form can be described by the equation (Berg 1982)

1 $$ J = - D\frac{\partial c}{\partial x} + v_{\text{d}} c, $$

where *c* is the species concentration, *x* is the spatial coordinate, and *v*_d_ is the drift velocity, obtained from the Langevin equation (Risken 1989)

2 $$ {{ma}} = - fv + F + \xi (t), $$

where *f* is the damping coefficient, *F* is the external force, and *ξ* is the random force. Assuming a high-friction limit (Risken 1989), i.e., that the term *ma* is small compared with other terms in (2), and calculating the average velocity (the drift velocity), one obtains

3 $$ v_{\text{d}} = \frac{F}{f}. $$

In case of a force generated by an electric potential, the following relation holds for *F* (Griffiths 1999):

4 $$ {F = - q\frac{{{\text{d}}\varphi }}{{{\text{d}}x}} = - ze\frac{{{\text{d}}\varphi }}{{{\text{d}}x}}}, $$

where *q* is the ionic charge described as a multiple *z* of the elementary charge *e*. 

To obtain the drift velocity one must substitute (4) into (3), taking into account the Einstein relation for the diffusion coefficient (Berg 1982) (i.e., *D* = *kT*/*f*, where *D* is the diffusion coefficient, *k* is the Boltzmann constant, and *T* is temperature). The final result reads

5 $$ {v_{\text{d}} = - \frac{Dze}{{kT}}\frac{{{\text{d}}\varphi }}{{{\text{d}}x}} = - \frac{DzF}{RT}\frac{{{\text{d}}\varphi }}{{{\text{d}}x}}}, $$

where *F* is the Faraday constant and *R* is the gas constant. Concluding, the flux reads

6 $$ J = - D\frac{\partial c}{\partial x} - \frac{DzFc}{RT}\frac{{{\text{d}}\varphi }}{{{\text{d}}x}}. $$

Constructing a mass balance equation leads to the Nernst–Planck equation (Coalson and Kurnikova 2007; Kurnikova et al. 1999; Rubinstein 1990)

7 $$ {\frac{\partial c}{\partial t} = - \frac{\partial }{\partial x}J = \frac{\partial }{\partial x}D\left[ {\frac{\partial c}{\partial x} + \frac{zFc}{RT}\frac{{{\text{d}}\varphi }}{{{\text{d}}x}}} \right]}. $$

Considering a fixed potential *φ*, this equation allows the evolution of the charge concentration to be calculated. However, a change in the charge concentration alters the electric potential, which needs to be recalculated to account for the change. Therefore, Eq. (7) must be supplemented by an equation for the electric potential *φ*, i.e., the Poisson equation (Rubinstein 1990; Griffiths 1999)

8 $$ {\frac{{{\text{d}}^{ 2} \varphi }}{{{\text{d}}x^{ 2} }} = - \frac{\rho }{\varepsilon }}, $$

where *ε* is the dielectric constant of the material (the product of the relative electric permittivity and the vacuum permittivity) and *ρ* is the charge concentration, which can be obtained from the molar concentration after multiplying by the Faraday constant and *z* as

9 $$ {\frac{{{\text{d}}^{ 2} \varphi }}{{{\text{d}}x^{ 2} }} = - \frac{zFc}{\varepsilon }}. $$

Because the cellular environment is populated by multiple ionic species, the above equations should be generalized to the case of many ions. In this way, one ends up with *N* diffusion equations of type (7) for each ionic species and one Poisson equation that takes into account all of the ions present in the environment, thus

10 $$ {\frac{{\partial c_{i} }}{\partial t} = - \frac{\partial }{\partial x}J_{i} = \frac{\partial }{\partial x}D\left[ {\frac{{\partial c_{i} }}{\partial x} + \frac{{z_{i} Fc_{i} }}{RT}\frac{{{\text{d}}\varphi }}{{{\text{d}}x}}} \right],\quad i = 1 ,\ldots ,N}, $$

11 $$ {\frac{{{\text{d}}^{ 2} \varphi }}{{{\text{d}}x^{ 2} }} = - \sum {\frac{{z_{i} Fc_{i} }}{\varepsilon }} }. $$

Equations (10, 11) set the theoretical framework for the present research.

### The data for the PNP model

To solve (10, 11) one must supplement them by the required parameters, including the ionic diffusion coefficients, the dielectric constant, and ionic concentrations. The main ion types present in a cell are K^+^ and Na^+^ ions. The cell’s negative charge originates mainly from proteins and HCO_3_^−^. The corresponding concentrations are presented in Table 1.

**Table 1:** Intracellular concentrations of the charge carriers

Ion	Intracellular concentration (mM)
Na^+^	12
K^+^	139
Proteins^−^	138
HCO_3_^−^	12

After Lodish et al. (2008)

As can be seen, the charge concentrations (which should in general be supplemented by the minor Cl^−^ and Mg^2+^ concentrations) reduce almost to zero. This finding is consistent with the estimate of a capacitive charge held by the cell membrane. The specific capacitance of the cell membrane can be estimated as *C*_0_ = 1 μF cm^−2^ (Neher and Sakmann 1995), which results in a capacitance of a spherical cell with radius set to *R* = 16 μm (the size of a Mat-Ly-Lu cell, rounded to a power of 2) equal to *C* = 4*πR* ^2^ *C*_0_ = 32 pF. This capacitance at a voltage of *U* = −90 mV charges the membrane with *n* = *CU*/*e*/*N*_a_3/4/*π*/*R* ^3^ = 0.002 mM of (monovalent) negative charge (*N*_a_ being the Avogadro number).

To simplify the model, I consider only charges due to proteins and Na^+^, K^+^, and Ca^2+^ ions, adjusting the protein charge to have the net charge of the cell consistent with the capacitive charge. The first two ionic species constitute most of the ions relevant to the process, and the Ca^2+^ ions, which are at very low concentration (<0.0002 mM; Lodish et al. 2008), are included for predicting calcium dynamics, having a minor effect on the electric potential distribution.

The diffusion coefficients of the intracellular ions are given in Table 2. The proteins are assumed to be immobile in the first approximation. It can be noticed that the diffusion coefficient for the calcium ions is larger than for the entering sodium ions, allowing effective repulsion of calcium ions rather than their being “overtaken” by the sodium.

**Table 2:** Diffusion coefficients of the intracellular ions

Ion	Diffusion coefficient
Na^+^	1.23 × 10^−10^ m^2^ s^−1^ (Abelson 1949)
K^+^	17.3 × 10^−10^ m^2^ s^−1^ (Hodgkin 1952)
Ca^2+^	5.3 × 10^−10^ m^2^ s^−1^ (Donahue 1987)

The dielectric constant is set close to the value in water, i.e., *ε*_0_ *ε*_r_ = 10^−9^ F m^−1^.

The boundary conditions for the Poisson equation are set to be *φ*(0) = 0 mV, *φ*(*L*) = −10 mV, where *L* = 32 μm is the cell length. The voltage difference is set to reflect the electric field of 3 V cm^−1^, as used in Djamgoz et al. (2001). The boundary conditions for the Nernst–Planck equation are essentially set to zero flux at the boundaries (a special procedure is introduced to handle VGSC opening, as described below).

The initial condition for the Nernst–Planck equation is set to be the (unphysical) uniform distribution. To obtain the real ionic distribution, the Nernst–Planck (NP) equation is solved for 50 μs (after which time the solution is essentially indistinguishable from a stationary solution of the NP equation). After that, a current pulse is applied for 1 ms. The value of this current is set to depolarize the membrane to 0 mV; i.e., at each of the *N*_c_ time steps of the simulation corresponding to the interval of 1 ms, a sodium concentration of 0.002 mM *Nx*/*N*_c_ is injected at *x* = 0 [keeping reflecting boundary conditions for the solution of the partial differential equation (PDE); *N* *x* = 32 is the number of grid points].

To investigate whether the assumption of a constant-current model of the VGSC activity limits the accuracy of the predictions (the actual sodium current has a pronounced spike), I modeled its action by a Dirac pulse. The conclusions were qualitatively similar, and the concentration changes at the cell boundaries were even more pronounced. However, since the real VGSC operates within about 1 ms, and not the simulation time step of 10 ps, I present the results for the constant-current model of sodium inflow. The details of the numerical procedures applied to the solution of model equations are described shortly in the "Appendix".

## Results and discussion

**Fig. 4:**
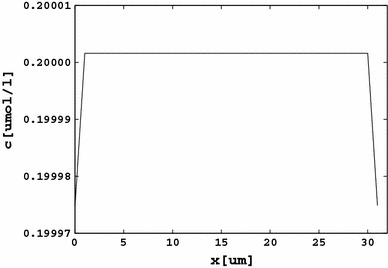
An example of the rest-state distribution of ions. The calcium distribution is shown. Due to the negative membrane voltage in the rest state, the regions close to the membrane walls are depleted of ions. Similar plots can be obtained for Na^+^ and K^+^

Figure 4 displays the rest-state distribution of the Ca^2+^ concentration. A similar chart can be obtained for the Na^+^ and K^+^ ions, because in the rest state the cell exhibits a negative membrane potential and a negative intracellular charge. The unbalanced charge sticks to the membrane within the Debye length (Rubinstein 1990), contributing to a gradient in the ionic concentration. The Debye length in a cellular environment is of the order of 0.7 nm (Małysiak and Grzywna 2009), which cannot be directly reflected by the results on a lattice spacing of 1 μm, and a smaller spacing is impossible to apply due to computer time limitations. The boundary concentration change shown in the chart is therefore averaged over 1 μm, and one should bear in mind that it is expected to be 1 μm/0.7 nm = 1,429 times larger within the Debye layer where the concentration can reach a value being 18 % smaller than it is in the bulk concentration.

**Fig. 5:**
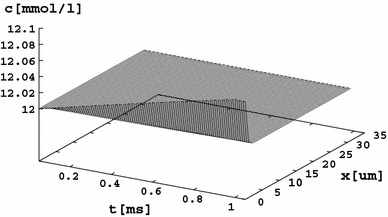
The concentration of sodium ions during the activation of the sodium channels. This process is modeled by a constant current reflected by an almost linear slope of the concentration at *x* = 0

Starting at rest conditions, the Na^+^ ions are injected into the cell domain, mimicking inflow at constant current, as shown in Fig. 5. In this figure one can see a linear increase in the Na^+^ concentration at *x* = 0, corresponding to the model of a constant sodium current. At this time resolution, diffusive effects are too slow to manifest and the concentration changes are linear with a slope reflecting the value of the current.

**Fig. 6:**
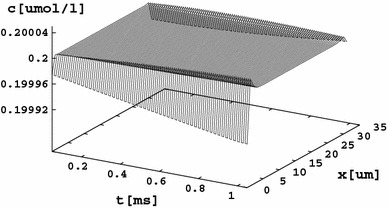
The Ca^2+^ concentration immediately reacts to the changes in sodium concentration. The concentration at *x* = 0 becomes decreased, and the concentration at *x* = 32 μm increases. The interaction is very stiff, and relaxation effects are not seen in this model (although they can be seen after a Dirac sodium pulse at sufficient time resolution). Similar behavior is observed for the K^+^ concentration distribution

**Fig. 7:**
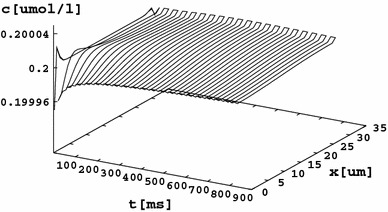
Calcium concentration distribution for a longer time period. The asymmetric concentration change remains for a long time

The Ca^2+^ concentration (as well as the K^+^ concentration) reacts immediately to the increase in Na^+^ at *x* = 0, as shown in Fig. 6. The injected Na^+^ ions repel the Ca^2+^ ions from the cathodal to the anodal side of the cell. This asymmetry holds even after time sufficient for diffusion to take place (Fig. 7).

The overall changes in the calcium concentration (Figs. 6, 7) seem to be small, but recalling the fact that they occur within the Debye length one can expect a change reaching a value of up to 57 % of the bulk concentration, which supports the view that the inflow of Na^+^ ions after cathodal depolarization may increase the anodal Ca^2+^ concentration (at the cost of the cathodal concentration). Such asymmetry may drive directional actin polymerization. In addition, as noted in the “Introduction,” the action of the VGSCs may decrease the pericellular pH (Carrithers et al. 2007), which further speeds up actin polymerization (Wang et al. 1989) and can cause Ca^2+^ uptake (or a reduction in release) by internal stores (Willoughby et al. 2001). These effects would enhance the cathodal direction of galvanotaxis; however, after the whole membrane is depolarized, they could stop working. On the other hand, the asymmetry in the electrically repelled calcium should hold even after the depolarization reaches the anodal part, because the Ca^2+^ ions cannot go back to the cathodal part, which is already rich in positive charges.

The presented analysis fits very well with the behavior of the weakly metastatic AT-2 cells. These cells do not exhibit cathodal galvanotaxis because they do not upregulate VGSCs to contribute to the Ca^2+^ repulsion on the cathodal side. Instead, the internal Ca^2+^ ions (and other cations) drift in the applied DC field to increase the concentration on the cathodal side (Fig. 3), resulting in weakly directed motion with anodal orientation. The theory could, however, be questioned by stating that the leakage influx of the Ca^2+^ ions leads to anodal accumulation of Ca^2+^, as proposed in Cooper and Keller (1984). This requires deeper discussion.

Taking into account the leak Ca^2+^ permeability of a lipid membrane (between 8 × 10^−15^ cm/s and 2.5 × 10^−11^ cm/s; Zeng et al. 1997) and considering a cell with radius *r* = 16 μm, one can calculate the currents corresponding to an intracellular Ca^2+^ concentration of 0.2 μM and extracellular concentration of 1.8 mM (Lodish et al. 2008). They range between 8.92 × 10^−20^ A and 2.78 × 10^−16^ A. Fitting the conductance according to the relation *I* = *g*_Ca,leak_(*U* – *U*_Ca_), where *U* is the rest membrane potential and *U*_Ca_ is the Nernst potential for Ca^2+^, and normalizing to the cell’s surface, one obtains *g*_Ca,leak_ varying between 13.2 fS/cm^2^ and 41.24 pS/cm^2^. With these data it is possible to calculate the increment of the leak Ca^2+^ current, associated with a DC field, on a model cubic cell with side length of 26 μm (the volume of such a cell being equal to the volume of the considered cells). With a DC field of 3 V/cm, the anodal membrane hyperpolarizes by about 5 mV, which results in a current change between 4.46 × 10^−22^ A and 1.39 × 10^−18^ A. This current could lead to accumulation of Ca^2+^ provided that the cytoplasmic current is lower and cannot account for such contribution. The cytoplasmic current can be estimated by considering the membrane resistance after Kager et al. (2000), equal to 75 ± 25 MΩ for a cell with surface of 1,686 μm^2^. The surface of the cubic face of the cubic cell is 676 μm^2^, and consequently, the membrane resistance of this face is higher, i.e., 187 MΩ. The resistance of the cubic cell’s cytoplasm is taken as 240 kΩ after Stinstra et al. (2005), who reported cytoplasm conductance of 0.16 S/m. Calculating the voltage divided across the membrane–cytoplasm–membrane resistances, one obtains a voltage drop for the cytoplasm of 12.8 μV. By Eq. (5), this predicts a cytoplasmic calcium current of 0.52 fA, larger than the increase in the leak influx. This indicates that the cytoplasm layers close to the membrane will actually become depleted of Ca^2+^.

The anodal leak accumulation of Ca^2+^ can however occur in some conditions. Such conditions may arise in some cells, for example, muscular cells, which are equipped with Ca^2+^ leak channels (Obejero-Paz et al. 1998). In this case, the rest-state leak current can take large values, of the order of 0.36 pA, which results in conductance of 0.21 μS/cm^2^ (A7r5 cell diameter ~16 μm), giving membrane current contributions due to the DC field too large to be compensated by the cytoplasmic current (7.2 fA). In such a case, the concentration of Ca^2+^ would increase locally (probably at the cost of K^+^ ions to keep the voltage unaltered) until the PMCA pump activates (due to the increased [Ca^2+^]) to a level sufficient to counterbalance this leak.

To discuss how the proposed mechanism fails to describe the VGSC-dependent anodal (not cathodal) galvanotaxis of MDA-MB-231 breast cancer cells (Fraser et al. 2005; Isbilen et al. 2006), one needs to consider mechanisms other than cathodal repulsion of Ca^2+^. One of the explanations could be a possible action of the VGCCs, but the literature states that the breast cancer cells do not possess VGCCs (Roger et al. 2004). Second, the breast cancer upregulates the plasma membrane Ca^2+^ (PMCA) pumps (Carafoli and Brini 2007; Monteith et al. 2007). Such pumps serve to decrease the intracellular calcium within the cell and could make the calcium concentration insufficient to influence the gelsolin-based blocking of actin polymerization, leaving only the pH dependency resulting from the VGSC activation (Wang et al. 1989; Carrithers et al. 2007). This, however, would imply cathodal galvanotaxis and cannot explain the anodal direction of motion. A final option could be that the NCX exchanger enters into the action [there is no literature evidence that it is absent in MDA-MB-231, and a good suggestion of its presence may be found in Winnicka et al. (2007)]. Such an exchanger could stop removing calcium from the cathodal side after depolarization [intracellular Na^+^ blocks the exchanger (Hilge et al. 2007)], opposing the repulsive effect in the decrease of the cathodal Ca^2+^ concentration.

## Conclusions

In this work, I show that the action of the VGSCs can cause repulsion of calcium ions from the cathodal side of the cell to the anodal side. In this way, an asymmetry in the calcium concentration can arise close to the membrane. It seems that such asymmetry is just what is needed to explain the VGSC-dependent cathodal galvanotaxis, which requires depletion of calcium on the cathodal side to cause actin polymerization and enrichment on the anodal side to cause myosin contraction.

What is important is that the presented theory requires a time delay between the depolarization events on the cathodal and on the anodal side of the cell. Such a time delay can also be used to propose directionality in other mechanisms acting on calcium dynamics, such as VGSC-mediated pH-induced internal store activity or facilitation of actin polymerization, or (in the case of cells that have them) in the operation of NCX exchangers.

The asymmetry in the calcium distribution remains even after diffusion starts to manifest, which suggests that the effect may hold for a longer period and may accumulate in subsequent depolarization cycles. It is not expected to be destroyed by the proceeding depolarization of the anodal part of the membrane, since the cathodal part already contains a large (repulsive) charge by that time, preventing any acceptance of additional charge. The asymmetry, in case of a real cell, can show even larger values of calcium concentration difference, because the sodium current reveals a spike. All this supports the qualitative model of galvanotaxis sketched in this paper, where the interplay between sodium and calcium ions is taken as the main contribution.

Importantly, the model not only explains the galvanotaxis of the highly metastatic prostate Mat-Ly-Lu cells but also accounts for the reduced mobility of AT-2 cells, and leaves enough space for the anodal galvanotaxis of breast cancer cells.

## Appendix: the solution method of the NP equation

The Nernst–Planck equation is a nonlinear drift–diffusion equation, which causes stability problems during numerical integration. To work around this problem, I have taken the approach of an explicit scheme with stabilization method, i.e., the explicit upwind scheme (Ni et al. 1998) applied to the drift term of the equation. The upwind scheme takes the following approximation to the first-order derivative of concentration:

$$ {\frac{{{\text{d}}c}}{{{\text{d}}x}} \approx \left\{ {\begin{gathered} \left( {c\left[ {x + h} \right] - c\left[ x \right]} \right)/h:\quad \frac{{{\text{d}}\varphi }}{{{\text{d}}x}} \ge 0\\ \left( {c\left[ x \right] - c\left[ {x - h} \right]} \right)/h:\quad \frac{{{\text{d}}\varphi }}{{{\text{d}}x}} < 0\\ \end{gathered} } \right\}}, $$

where the potential derivative is taken as a central difference. The time step for the simulation depends upon the gradient of the potential and initially was taken to be equal to 10 ps, after 1.1 ms being increased to 100 ps (further increases are possible but were not used in this research).

The second-order derivative was calculated as a central difference in a standard way. This method allowed results to be obtained for reasonable dielectric constants $(\epsilon_r=100)$ while alternative approaches such as the Crank–Nicolson implicit scheme became unstable at dielectric constant values several orders of magnitude weaker.
